# Programmed death‐ligand 1 expression in gastric cancer: correlation with mismatch repair deficiency and HER2‐negative status

**DOI:** 10.1002/cam4.1502

**Published:** 2018-04-19

**Authors:** Lei Wang, Qiongyan Zhang, Shujuan Ni, Cong Tan, Xu Cai, Dan Huang, Weiqi Sheng

**Affiliations:** ^1^ Department of Pathology Fudan University Shanghai Cancer Center Shanghai 200032 China; ^2^ Department of Oncology Shanghai Medical College Fudan University Shanghai 200032 China

**Keywords:** Gastric cancer, HER2, mismatch repair deficiency, programmed death‐ligand 1

## Abstract

Gastric cancer (GC) is one of the most common malignancies. Immunotherapy is a promising targeted treatment. The immune regulatory programmed death‐1 (PD‐1)/programmed death‐ligand 1 (PD‐L1) axis has been used as a checkpoint target for immunotherapy. Currently, considerable discrepancies exist concerning the expression status of PD‐L1 and its prognostic value in GC. We aimed to evaluate the expression rates of PD‐L1 in GC, and further assess its relationship with mismatch repair (MMR), and human epidermal growth factor receptor 2 (HER2) status. We retrospectively collected 550 consecutive cases of GC in Fudan University Shanghai Cancer Center from 2010 to 2012. PD‐L1, MMR protein, and HER2 status were detected by immunohistochemistry (IHC). Fluorescence in situ hybridization was further used in HER2 IHC 2+ cases. Cases with at least 1% membranous and/or cytoplasmic PD‐L1 staining in either tumor cells (TCs) or tumor‐infiltrating immune cells (TIICs) were considered as PD‐L1 positive. The correlation between clinicopathological parameters, HER2, MMR, and PD‐L1 expression status was determined using chi‐squared tests. About 37.3% cases (205/550) showed PD‐L1 expression in TCs and/or TIICs. 17.3% cases (95/550) showed PD‐L1 expression in TCs, 34.5% (190/550) cases showed PD‐L1 expression in TIICs. There were 45 deficient MMR (dMMR) cases (8.2%), which showed higher rates of PD‐L1 expression compared with MMR‐proficient carcinomas (60.0% vs. 35.2%, *P *= 0.001). HER2 was positive in 66 (12.0%) cases. The expression of PD‐L1 occurred more frequently in HER2‐negative group than HER2‐positive cohorts (39.0% vs. 24.2%, *P *= 0.020). The survival analysis revealed that PD‐L1 was not associated with prognosis. This study evaluated the association between the PD‐L1 expression and a specific subgroup (dMMR and HER2‐negative) in a large Asian cohort of GC. GC patients with dMMR and HER2‐negative status exhibited higher PD‐L1 expression rates. Our finding indicated that MMR and HER‐2 status might be potential biomarkers for anti‐PD‐L1 therapy.

## Background

Gastric cancer (GC) is one of the most prevalent cancer in the world 1. Although the incidence and mortality of GC have been decreasing over the past several decades, GC remains one of the most common cancers in China. There are still lots of GC patients diagnosed at an advanced or inoperable stage. Chemotherapy can improve the overall survival rate of these patients, however, there is no precise treatment for these patients. Given the need for more efficacious therapy for the advanced stage patients, target therapies are becoming a hot study area. For example, Trastuzumab is an effective and well‐tolerated drug for advanced stage GC patients 2. However, the benefit of Trastuzumab is limited in human epidermal growth factor receptor 2 (HER2) positive patients. Yet HER2 overexpression is reported in only about 12% advanced GC patients in China 3. The treatment remains a big challenge for the vast of HER2 negative GC patients.

Immunotherapy using immune checkpoint inhibitors is a rapidly growing modality for the treatment of human cancers 4. The immune regulatory programmed death‐1 (PD‐1)/programmed death‐ligand 1 (PD‐L1) axis can induce immune inhibitory signaling to activated T cells and destroy the antitumor immune response, which has been used as an immune checkpoint target for immunotherapy in various malignancies. PD‐1/PD‐L1 blockers can recover the native antitumor function of T cells 5. PD‐1 expression was found in tumor‐infiltrating lymphocytes (TILs) 5, 6. And PD‐L1 is expressed on tumor cells (TCs) and some immune cells (including lymphocytes, macrophages, and dendritic cells) 7, 8, 9. Immunotherapy with PD‐1 or PD‐L1 antibodies has been revealed to be effective in malignant melanomas, non‐small lung cancer, renal cell carcinomas, and bladder carcinomas 10, 11. In colorectal cancer (CRC), it has been demonstrated that patients with mismatch repair deficiency (dMMR) are good responders to anti‐PD‐1/PD‐L1 immunotherapy, which indicates the mismatch repair (MMR) status can be used as a potential candidate for anti‐PD‐1/PD‐L1 therapy in CRCs 12. In the GC treatment research field, anti‐PD‐1/PD‐L1 therapy also showed promising antitumor activity 13, 14. However, studies addressed the biomarkers of anti‐PD‐1/PD‐L1 therapy in GC are rare. Besides, considerable varieties exist concerning the expression rate of PD‐L1 in GC. The relationship between PD‐L1 expression and the status of MMR proteins or HER2 expression also needs to be understood profoundly. So this study evaluated the expression rate of PD‐L1 and further explored its correlation with MMR proteins or HER2 expression in a large Asian cohort of GC.

## Methods

### Tumor specimens and clinical data collection

The study was approved by The Clinical Research Ethics Committee of Fudan University Shanghai Cancer Center. Written informed consent was obtained from all participants included in the study. Five hundred and fifty cases of surgically resected gastric cancer were collected from the files of Department of Pathology, Fudan University Shanghai Cancer Center. All the patients had not undergone previous chemotherapy treatment. All the cases were reviewed by two pathologists and the histological diagnoses were confirmed without discrepancy. Clinical findings, including age, gender, tumor location and size, therapy, and clinical outcome were obtained from the medical record, pathology report, or discharge summary. The follow‐up information was conducted via medical records plus telephone interview, and the following information was obtained as follows: follow‐up duration, disease‐free survival (DFS), and overall survival (OS).

### Immunohistochemical (IHC) staining and evaluation

IHC was performed on 4 *μ*m‐thick tissue sections with the automated immunohistochemical stainer (Ventana, Tucson, AZ). The primary antibodies used in the study included anti‐PD‐L1 (Clone 28‐8, Abcam, Cambridge, MA), anti‐MLH1 (Clone M1, Ventana), anti‐PMS2 (Clone EPR3947, Ventana), anti‐MLH2 (Clone G219‐1129, Ventana), anti‐MLH6 (Clone 44, Ventana), and anti‐ erbB‐2 (HER2) (Clone 4B5, Ventana). Omission of primary antibody and substitution by non‐specific immunoglobins were used as negative controls. The appropriate specificity and sensitivity of the antibody against PD‐L1 staining were determined using human placenta as the positive control. Appropriate positive controls were run concurrently for all antibodies tested. PD‐L1 expression showed membranous staining and/or cytoplasmic staining. The proportion of immunostained cells was evaluated among tumor cell and tumor‐infiltrating immune cells. The patients with at least 1% PD‐L1 staining of tumor cells or immune cells were considered positive. The cases showed preserved nuclear expression of 4 MMR proteins were considered MMR‐proficient (pMMR). HER2 expression was graded using a score scale of 0 to 3 15.

### Fluorescence in situ hybridization and evaluation

Fluorescence in situ hybridization (FISH) studies were performed on HER2 IHC2+ cases. Representative sections were incubated in a humidified chamber using HER2 Probe (HER2, orange; Chromosome 17 centromere, green) according to the manufacturer's protocol (Vysis HER2/CEP 17 FISH Probe Kit, Vysis, Abbott, Des Plaines, IL). For gene amplification assessment, the total number of HER2 and chromosome 17 signals was counted in at least 10 high power fields. The HER2/chromosome 17 ratios which was equal or greater than 2.2 was considered as gene amplification.

### Statistical analysis

The chi‐squared and the Fisher test were used to test the difference between groups. The survival difference between groups was assessed by the Kaplan–Meier method. Differences were considered to be significant at *P* < 0.05. SPSS (Chicago, IL) version 18.0 was used to analyze all data.

## Results

### PD‐L1 expression in gastric cancers and their correlation with clinicopathological features

**Figure 1:**
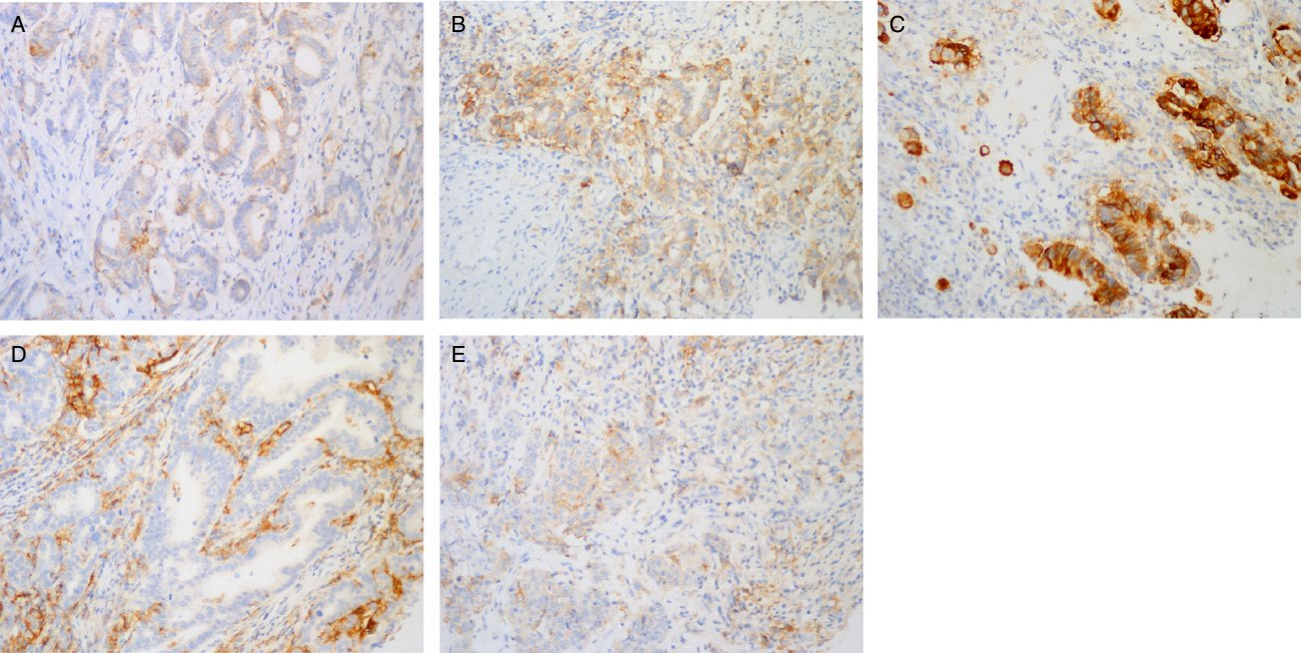
Representative images of PD‐L1 immunostaining. PD‐L1 was immunostained on the membrane and/or the cytoplasm of the tumor cells with variable intensities: (A) weak (score 1), (B) moderate (score 2), (C) strong (score 3). (D) PD‐L1 was immunostained only in tumor‐infiltrated immune cells. (E) PD‐L1 expression was detected in both tumor cells and tumor‐infiltrating immune cells.

PD‐L1 was detected in the tumor cells and/or tumor‐infiltrating immune cells with variable intensities and proportions, but not in non‐neoplastic gastric epithelium (Fig. 1). 95 of 550 cases (17.3%) showed PD‐L1 expression in TCs, while 190 cases (190/550, 34.5%) had positive PD‐L1 staining in TIICs. A total of 205 cases (205/550, 37.3%) showed PD‐L1 expression in TCs and/or TIICs. PD‐L1 expression was observed more frequently in GCs occurred in those older patients (≥60 years) (TCs *P *=* *0.001, TIICs *P *=* *0.004, TCs and/or TIICs *P *=* *0.001), with bigger size (≥5 cm) (TCs *P *<* *0.001, TIICs *P *=* *0.032, TCs and/or TIICs *P *=* *0.035) and solid‐type histological features (TCs *P *<* *0.001, TIICs *P *<* *0.001, TCs and/or TIICs *P *<* *0.001). PD‐L1 expression was less common in the GCs with submucosal invasion (TCs *P *=* *0.049, TIICs *P *=* *0.001, TCs and/or TIICs *P *<* *0.001). And GCs in stage II, stage III, and IV showed higher PD‐L1 expression than GCs in stage I (TCs *P *=* *0.016, TIICs *P *<* *0.001, TCs and/or TIICs *P *<* *0.001). Clinicopathological characteristics of GC are summarized in Table 1.

**Table 1:** Clinicopathological characteristics and their correlation with PD‐L1 expression

	Total valid *n* = 550	PD‐L1 in TCs			PD‐L1 in TIICs			PD‐L1 in TCs and/or TIICs		
		Negative *n* = 30	Positive *n* = 541	*P*‐value	Negative *n* = 360	Positive *n* = 190	*P*‐value	Negative *n* = 97	Positive *n* = 474	*P*‐value
Gender										
Male (%)	375	307 (82)	68 (18)	0.434	250 (67)	125 (33)	0.382	238 (64)	137 (36)	0.600
Female (%)	175	148 (85)	27 (15)		110 (63)	65 (37)		107 (61)	68 (39)	
Age (years)										
<60 (%)	278	245 (88)	33 (12)	0.001	198 (71)	80 (29)	0.004	193 (69)	85 (31)	0.001
≥60 (%)	272	210 (77)	62 (23)		162 (60)	110 (40)		152 (56)	120 (48)	
Location										
Cardia (%)	147	118 (80)	29 (20)	0.069	92 (63)	55 (37)	0.579	85 (58)	62 (42)	0.408
Corpus (%)	200	162 (81)	38 (19)		135 (68)	65 (32)		130 (65)	70 (35)	
Antrum (%)	188	165 (88)	23 (12)		125 (67)	63 (33)		122 (65)	66 (35)	
Diffuse (%)	15	10 (67)	5 (33)		8 (53)	7 (47)		8 (53)	7 (47)	
Size										
<5 cm (%)	404	349 (86)	55 (14)	<0.001	275 (68)	129 (32)	0.032	264 (65)	140 (35)	0.035
≥5 cm (%)	146	106 (73)	40 (27)		85 (58)	61 (42)		81 (55)	65 (45)	
Lauren										
Intestinal (%)	231	185 (80)	46 (20)	<0.001	141 (61)	90 (39)	<0.001	129 (56)	102 (44)	<0.001
Diffused (%)	210	193 (92)	17 (8)		168 (80)	42 (20)		166 (79)	44 (21)	
Mixed (%)	87	70 (81)	17 (19)		49 (56)	38 (44)		48 (55)	39 (45)	
Solid‐type (%)	22	7 (32)	15 (68)		2 (9)	20 (91)		2 (9)	20 (91)	
T‐Category										
T1 (%)	79	73 (92)	6 (8)	0.049	66 (84)	13 (16)	0.001	66 (84)	13 (16)	<0.001
T2 (%)	68	55 (81)	13 (19)		40 (59)	28 (41)		37 (54)	31 (46)	
T3 + T4 (%)	403	327 (81)	76 (19)		254 (63)	149 (37)		242 (60)	161 (40)	
LN metastases										
Negative (%)	175	142 (81)	33 (19)	0.502	105 (60)	70 (40)	0.066	102 (58)	73 (42)	0.141
Positive (%)	375	313 (83)	62 (17)		255 (68)	120 (32)		243 (65)	132 (35)	
M‐Category										
M0 (%)	538	445 (83)	93 (17)	1.000	352 (65)	186 (35)	1.000	337 (63)	201 (37)	1.000
M1 (%)	12	10 (83)	2 (17)		8 (67)	4 (33)		8 (67)	4 (33)	
TNM stage										
I (%)	106	96 (91)	10 (9)	0.006	83 (78)	23 (22)	<0.001	82 (77)	24 (23)	<0.001
II (%)	136	102 (75)	34 (25)		66 (48)	70 (52)		61 (45)	75 (55)	
III/IV (%)	308	257 (83)	51 (17)		211 (68)	97 (35)		202 (65)	106 (35)	

LN, lymph node; TCs, tumor cells; TIICs, tumor‐infiltrating immune cells.

### dMMR gastric cancers tend to show positive PD‐L1 expression

**Figure 2:**
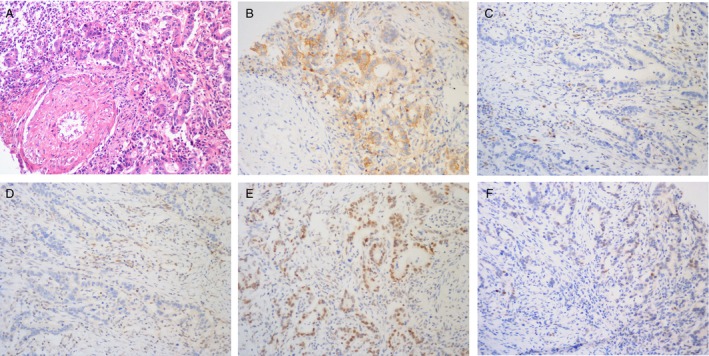
Deficient MMR GCs tend to show positive PD‐L1 expression. In this cases (A, HE), PD‐L1 expressed mainly in the tumor cells (B). MLH1 and PMS were negative (C, D), and MSH2 and MSH6 are positive (E, F).

dMMR could be detected in 45 patients (8.2%) in our cohort. As shown in Table 2, 6.0% (33/550) and 6.2% (34/550) cases lost MLH1 and PMS2 expression, and only 0.2% (1/550) and 2.7% (15/550) cases did not show the expression of MSH2 and MSH6, respectively. PD‐L1 expression in TCs and/or TIICs was observed in 21 of 33 cases (63.6%) without MLH1 expression and in 22 of 34 cases (64.7%) without PMS2 expression, while in only 35.6% (184/517) cases with MLH1 expression and in 35.5% (183/516) cases with PMS expression (*P *=* *0.001, each, Table 2). For the only one MSH2 negative cases, PD‐L1 expression could not be detected. And 7 cases of 15 MSH6 negative cases (46.7%, *P *=* *0.446) present positive PD‐L1 staining (Table 2). Above all, GCs with dMMR showed higher rates of PD‐L1 expression compared with pMMR carcinomas (60.0% vs. 35.2%, *P *=* *0.001, Table 2, Fig. 2).

**Table 2:** The relationship between PD‐L1 expression and MSI status in gastric cancer

	Total valid	PD‐L1 in TCs			PD‐L1 in TIICs			PD‐L1 in TCs and/or TIICs		
		Negative	Positive	*P*‐value	Negative	Positive	*P*‐value	Negative	Positive	*P*‐value
MLH1										
Negative (%)	33	22 (67)	11 (33)	0.012	13 (39)	20 (61)	0.001	12 (36)	21 (64)	0.001
Positive (%)	517	433 (84)	84 (16)		347 (67)	170 (33)		333 (64)	184 (36)	
PMS2										
Negative (%)	34	22 (65)	12 (35)	0.004	13 (38)	21 (62)	0.001	12 (35)	22 (65)	0.001
Positive (%)	516	433 (84)	83 (16)		347 (67)	169 (33)		333 (64)	183 (36)	
MSH2										
Negative (%)	1	1 (100)	0 (0)	1.000	1 (100)	0 (0)	1.000	1 (100)	0 (0)	1.000
Positive (%)	549	454 (83)	95 (17)		359 (65)	190 (35)		344 (63)	205 (37)	
MSH6										
Negative (%)	15	14 (93)	1 (7)	0.487	8 (53)	7 (47)	0.317	8 (53)	7 (47)	0.446
Positive (%)	535	441 (82)	94 (18)		352 (66)	183 (34)		337 (63)	198 (37)	
MMR status										
MSI (%)	45	33 (73)	12 (27)	0.082	19 (42)	26 (58)	0.001	18 (40)	27 (60)	0.001
MSS (%)	505	422 (84)	83 (16)		341 (68)	164 (32)		327 (65)	178 (35)	

TCs, tumor cells; TIICs, tumor‐infiltrating immune cells.

### HER2 negative gastric cancers tend to show positive PD‐L1 expression

HER2 was positive in 66 (66/550, 12.0%) cases, among which 16 cases were positive for PD‐L1 (24.2%). However, in HER2‐negative group, 39.0% (189/484) of tumors were positive for PD‐L1 in TCs and/or TIICs. The expression rate of PD‐L1 in HER2 negative GCs was significantly higher than that in HER2 positive GCs (*P *=* *0.020, Table 3).

**Table 3:** The relationship between PD‐L1 expression and HER2 status in gastric cancer

	Total valid	PD‐L1 in TCs			PD‐L1 in TIICs			PD‐L1 in TCs and/or TIICs		
		Negative	Positive	*P*‐value	Negative	Positive	*P*‐value	Negative	Positive	*P*‐value
HER2 status										
Negative (%)	484	398 (82)	86 (18)	0.405	306 (63)	178 (37)	0.003	295 (61)	189 (39)	0.020
Positive (%)	66	57 (86)	9 (14)		54 (82)	12 (18)		50 (76)	16 (24)	

TCs, tumor cells; TIICs, tumor‐infiltrating immune cells.

### Survival analysis

**Figure 3:**
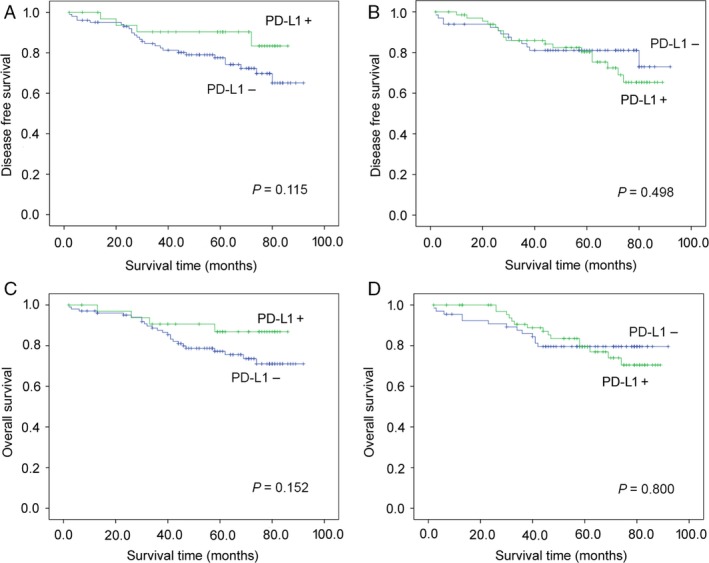
Kaplan–Meier plots of disease‐free survival and overall survival according to PD‐L1 expression in tumor cells (A, C) and tumor‐infiltrating immune cells (B, D) in stage II GC patients.

**Figure 4:**
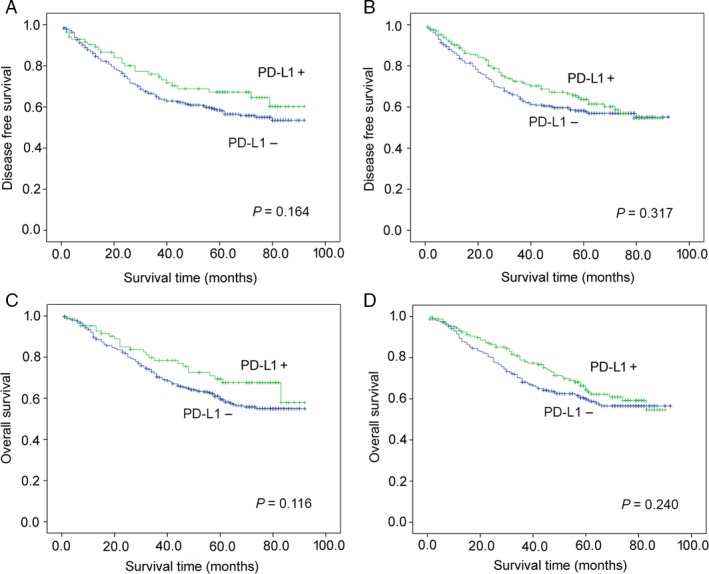
Kaplan–Meier plots of disease‐free survival and overall survival according to PD‐L1 expression in tumor cells (A, C) and tumor‐infiltrating immune cells (B, D) in stage III and IV GC patients.

The survival analyses of the 136 patients in stage II, the 444 patients in stage III and IV were presented in Figures 3 and 4, respectively. It turned out that PD‐L1 expression in TCs and/or TIICs was not correlated with the patients’ prognosis.

## Discussion

Our study was conducted to investigate the correlation between PD‐L1 expression and clinicopathological features of GC patients. This cohort included a considerable larger number of GC patients (*n* = 550) than most previous studies. In our study, the positive expression of PD‐L1 was observed in 37.3% cases. PD‐L1 was detected in 15% to 75% of GC patients according to previous literatures 16, 17, 18, 19, 20. We suppose varied antibodies, different tissue handling methods, and evaluating systems used to define PD‐L1 positivity may all lead to such a wide range of distinct expression rates. Besides, the intratumoral heterogeneity can be observed in PD‐L1 expression in GC tissue. We also detected the expression level of PD‐L1 using the tissue microarray (TMA) of this cohort. However, PD‐L1 was positive in only 17.1% cases (94/550) using TMA, which was much lower than that of using the whole tissue block (37.3%, 205/550) (κ* *= 0.52, *P *<* *0.001) (data was not shown). The reason for the difference in positivity rate of PD‐L1 was that PD‐L1 expression was heterogeneous, there is a chance that PD‐L1 positive tumor areas not be taken into TMA as tissue cores were drilled at random from the tissue blocks. In our study, in order to minimize the intratumoral heterogeneity, each case had two cores taken into TMA, one from the center and the other from the invasive front. It has recently been reported that PD‐L1 expression was frequently discordant between surgical specimens and matched biopsy specimens because of the heterogeneity of PD‐L1 expression 21. In fact, some GC patients are diagnosed at inoperable stages, only biopsies might be available for these patients. However, if the PD‐L1 positive tumor tissues were not sampled by biopsy, it might carry the risk of a false‐negative result. Therefore, the specific methods of PD‐L1 evaluation in biopsy need to be standardized. And the value of PD‐L1 as a predictive biomarker for anti‐PD‐L1 therapy in biopsy specimens might also need to be further discussed.

PD‐L1 expression has been observed in various tumors, and lots of studies demonstrated that PD‐L1 is a potential prognostic biomarker 20, 22, 23. However, the relationship between PD‐L1 expression and the prognosis remains controversial in GC. Many researches showed that PD‐L1 expression had a negative impact on patient survival 24, 25, 26, 27, but some studies indicated that PD‐L1 positivity was associated with favorable outcomes 16, 28. In our study, PD‐L1 expression in GC was related to some adverse clinicopathological characteristics, which was observed more frequently in advanced GCs occurred in the older patients and with bigger tumor size. Many studies reported that GC patients with dMMR had a better prognosis in comparison with pMMR patients 29, 30. However, GCs with dMMR showed higher rates of PD‐L1 expression compared with pMMR carcinoma. Its positive correlation with dMMR indicated that PD‐L1 expression in GC might play a beneficial role on prognosis, which was in contradiction with the results of PD‐L1 expression related to some adverse clinicopathological features. Therefore, in our study, no association between PD‐L1 expression and the prognosis was observed neither in stage II nor in stage III‐IV GC patients. In our opinion, these different conclusions reported in the literatures may be influenced by distinct research cohorts (limited number of patients or different clinical stages), different antibodies, and evaluation methods used to detect or define PD‐L1 positivity.

PD‐L1 expression might be as a predictive biomarker for anti‐PD‐1/PD‐L1 treatment. Because of the heterogeneity of PD‐L1 expression, surrogate biomarkers for PD‐L1 expression should be explored. It has been demonstrated that patients with mismatch repair deficiency are good responders to anti‐PD‐1/PD‐L1 immunotherapy in CRC patients 31. Our research showed that GC with dMMR showed higher rates of PD‐L1 expression. Considering these findings, anti‐ PD‐1/PD‐L1 immunotherapy might have more efficacies in dMMR GCs. Moreover, we found that positive PD‐L1 expression occurred significantly more often in HER2‐negative GCs, which might lead to a novel treatment strategy. As only HER2‐positive patients can benefit from Trastuzumab and other kind of anti‐HER2 drugs, whereas the proportion of HER2 overexpression in GC patients is about 15–22% 32, 33. In China, the positivity rate of HER2 is even lower, only 12% according to the data from 11 hospitals 3. Anti‐PD‐1/PD‐L1 immunotherapy might become a potentially new treatment for HER2 negative patients. However, reports focusing on the correlation between PD‐L1 and HER2 had conflicting conclusions. Oki et al. 34 found a positive relation between PD‐L1 and HER2 expression. On the contrary, the current study, as well as the work of Li et al. 27 indicated PD‐L1 expression was significantly associated with lower HER2 expression. Thus, future studies should be performed to clarify the relationship between PD‐L1 and HER2 expression.

All together, our research found that MMR deficiency and HER2‐negative status might be used as surrogate biomarkers for PD‐L1 expression. And anti‐PD‐1/PD‐L1 immunotherapy might be used as a potential candidate for GC patients with positive PD‐L1 expression, MMR deficiency, and negative HER2 status.

## Conclusions

In summary, this study evaluated the association between the PD‐L1 expression and a specific subgroup (dMMR and HER2‐negative) in a large Asian cohort of GC patients. Our study demonstrated that PD‐L1 expression in GC is significantly correlated with dMMR and HER2‐negative status. We also found that PD‐L1 expression was not related to patients’ prognosis. PD‐1/PD‐L1 checkpoint inhibitors might become a novel therapy strategy to HER2‐negative patients.

## Conflict of Interest

The authors declare that they have no conflict of interest.
